# Drug repurposing using meta-analysis of gene expression in Alzheimer’s disease

**DOI:** 10.3389/fnins.2022.989174

**Published:** 2022-11-10

**Authors:** Ha Young Jang, Jung Mi Oh, In-Wha Kim

**Affiliations:** ^1^Research Institute of Pharmaceutical Sciences, Seoul National University, Seoul, South Korea; ^2^College of Pharmacy, Seoul National University, Seoul, South Korea

**Keywords:** drug repositioning, gene expression, Alzheimer’s disease, meta-analysis, *in silico*

## Abstract

**Introduction:**

Alzheimer’s disease and other forms of dementia are disease that bring an increased global burden. However, the medicine developed to date remains limited. The purpose of this study is to predict drug repositioning candidates using a computational method that integrates gene expression profiles on Alzheimer’s disease and compound-induced changes in gene expression levels.

**Methods:**

Gene expression data on Alzheimer’s disease were obtained from the Gene Expression Omnibus (GEO) and we conducted a meta-analysis of their gene expression levels. The reverse scores of compound-induced gene expressions were computed based on the reversal relationship between disease and drug gene expression profiles.

**Results:**

Reversal genes and the candidate compounds were identified by the leave-one-out cross-validation procedure. Additionally, the half-maximal inhibitory concentration (IC50) values and the blood-brain barrier (BBB) permeability of candidate compounds were obtained from ChEMBL and PubChem, respectively.

**Conclusion:**

New therapeutic target genes and drug candidates against Alzheimer’s disease were identified by means of drug repositioning.

## Introduction

Alzheimer’s disease is the most common cause of dementia, a common neurodegenerative disease. Dementia is an escalating global burden disease, recently estimated to affect 57.4 million people worldwide as of 2019, increasing to 153.8 million by 2050 (GBD 2019 Dementia Forecasting Collaborators, 2022). Although the underlying mechanisms of Alzheimer’s disease remain not fully understood (Burns and Iliffe, 2009), currently Alzheimer’s disease is characterized by initial forgetfulness and cognitive decline that can affect behavior and the motor system finally (DeTure and Dickson, 2019). The presence of extracellular beta-amyloid deposition and intracellular neurofibrillary tangles consisting of hyperphosphorylated tau are the neuropathological hallmark features for an Alzheimer’s disease diagnosis (Long and Holtzman, 2019).

Drugs can be an important part of Alzheimer’s disease treatments. However, there is no treatment available to cure Alzheimer’s disease, only those that can treat the symptoms for a while or slow down their progression in some patients (Folch et al., 2018). The most commonly used medicines are cholinesterase inhibitors such as donepezil, galantamine, and rivastigmine, and an N-methyl-D-aspartate receptor antagonist, memantine (Scheltens et al., 2021). Despite the fact that aducanumab, a monoclonal antibody that reduces beta-amyloid deposits in the brain, was approved in an accelerated manner by the U.S. FDA in 2021 (Mullard, 2021), there is still some argument against its use (Whitehouse et al., 2022). The European Medicines Agency rejected this drug’s approval for the treatment of mild cognitive impairment due to Alzheimer’s disease and mild Alzheimer’s dementia (Mahase, 2021). Therefore, it remains necessary to discovery new drug targets and drugs against Alzheimer’s disease due to the ambiguity in efficient pathological targets or the adverse effects of previously used drugs.

Drug repositioning refers to the application of established drugs to new therapeutic indications (Langedijk et al., 2015), accelerating the development process due to the reductions in the time and costs involved in the progress of evaluating preclinical safety. Publicly available transcriptomic data generated from disease samples, as well as compounds, provide an opportunity for understanding the pathologies of diseases and the mechanisms of actions of drugs and for discovering new applications for existing drugs (Jarada et al., 2020). Recently, computational techniques using genome-wide gene expressions, especially chemical-induced or disease-specific differential gene expression levels, network analyses, or deep-learning approaches, have been applied for the identification of new therapeutic targets and drug repurposing (Lamb et al., 2006; Dudley et al., 2011; Chen et al., 2017; Advani and Kumar, 2021; Chyr et al., 2022).

Especially in relation to neurodegenerative diseases, which require long-term treatments in elderly patients, who frequently have comorbidities and take a multitude of medications, clinical trials are difficult. Consequently, the purpose of this study is to identify potential candidate drugs as a treatment for Alzheimer’s disease using a systematic computational drug repositioning method that combines disease-specific and drug-induced gene expression profiles.

## Materials and methods

### Collection of gene expression data on disease

Gene expression data related to Alzheimer’s disease were downloaded from the Gene Expression Omnibus (GEO) database established at the National Center for Biotechnology Information (NCBI) (Barrett and Edgar, 2006). The keywords were “Alzheimer’s disease” and the search results were limited to those published from 2017 to May of 2022, “*Homo sapiens*” as the organism, and “expression profiling by array” as the study type were used. These data, including the GEO accession number, summary, sample type, overall design, and platform were collected. The brain regions of the entorhinal cortex (EC), medial temporal gyrus (MTG) and temporal cortex (TC) were selected. The dataset containing healthy controls was included. If the Braak stages of the samples were available, the samples with Braak scores ≤3 from Alzheimer’s disease or the samples with Braak scores ≥3 from controls were removed from any further analysis (Patel et al., 2019). Gene expression data pertaining to brain tissues from patients with Alzheimer’s disease or controls were downloaded. The expression levels were transformed to logarithms and normalized. If multiple probes were assigned to one gene, the probe with the highest interquartile range was selected as the representative probe for that gene.

### Meta-analysis of gene expression on disease

To obtain a robust result, 10% of non-expressed genes based on mean intensities across the studies and 30% of non-informative genes based on variance were removed. The standardized mean rank method in MetaQC (Kang et al., 2012) was initially applied to conduct a gene expression quality check of each GEO dataset. Quantitative quality control (QC) measures including internal QC, external QC, accuracy QC, and consistency QC of genes or pathways and the standardized mean rank summary score for each dataset were assessed. All *P*-values were adjusted by Bonferroni correction. Additionally, a principal component analysis (PCA) biplot was applied to the visualization and helped determine the inclusion or exclusion of studies in the meta-analysis. The MetaDE package (Wang et al., 2012) was used to identify meta-differentially expressed genes (DEGs) between Alzheimer’s disease and the control. The *P*-values and combined effect sizes in the meta-analysis were calculated with a fixed effect model (Choi et al., 2003). This meta-gene expression levels were used as disease signatures for further analysis.

### Collection of gene expression data on compounds

Chemical compound-induced genome-wide transcriptional profiles were downloaded from the Library of Integrated Network-based Cellular Signatures (LINCS) (Koleti et al., 2018). Information regarding the cellular contexts, treatment time points, and treatment concentrations across multiple compounds was collected.

### Computation of reverse gene expression scores

Reverse gene expression scores were computed using the method described by Chen et al. (2017), and the description in the methods section partly reproduces their wording. Reverse scores of compounds indicate a reversal correlation between the DEGs associated with disease and compounds. An enrichment reverse score of gene expression associated with disease was computed based on the DEG rank. Additionally, drug-associated genes were ranked based on their expression levels as regulated by each compound from LINCS. Therefore, if the possibility of reversing the gene expression associated with the disease is higher, the negative value of the reverse score becomes lower. The result is such that each compound can have more than one reverse gene expression score. If multiple scores were computed for the one compound, summarized reverse scores of gene expression were calculated by weighted linear regression considering the experimental conditions, including the cell lines, concentrations, and treatment time points of the compounds. The reference condition established here used a concentration of 10 μM and treatment time of 24 h (Chen et al., 2017).

### Identification candidate compounds and reversed genes

Genes whose expression levels were reversed by a compound were determined by the leave-one-out cross-validation process to decrease over-fitting problems (Cheng et al., 2017). Upregulated genes were ranked toward the top, whereas downregulated genes were ranked toward the bottom. Each compound in turn was taken to be the test set, while the data for the remaining compounds served as the training set. Reversed genes by compound were then identified using the process described above. In all trials, false discovery rate-adjusted *P*-values of less than 0.05 were considered as indicative of reversal genes.

### Cell cytotoxicity

Bioactivity IC50 data using human brain tumor cells were downloaded from ChEMBL (Gaulton et al., 2017). Information on the units, molecular weights, cells, and organisms was collected. Compounds in ChEMBL were manually mapped with those in LINCS based on the IUPAC International Chemical Identifier (InChI). If multiple IC50 values were available for one compound, the corresponding median value was calculated.

### Prediction of permeability across the blood-brain barrier

The blood-brain barrier (BBB) permeability of the compounds in each case was predicted by the DeepPred-BBB model (Kumar et al., 2022) using their canonical simplified molecular input line entry system (SMILES). The SMILES strings were obtained from PubChem (Kim et al., 2021).

## Results

### Disease gene expression signatures

**FIGURE 1:**
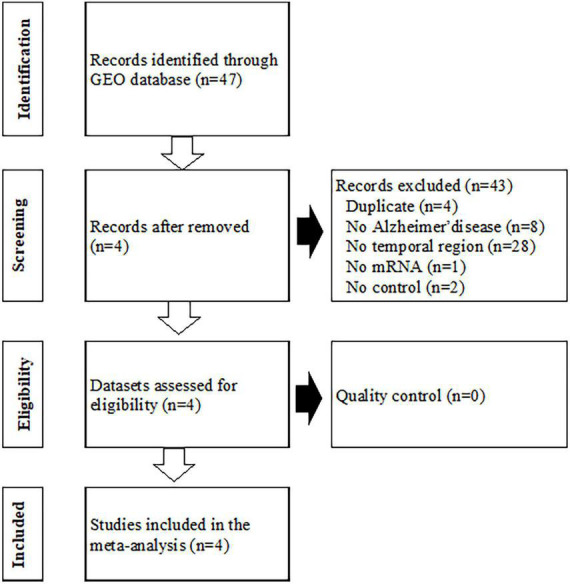
Flowchart of the process used to select gene expression datasets for the meta-analysis of Alzheimer’s disease. GEO, Gene Expression Omnibus.

Gene expression data for Alzheimer’s disease were searched for and download from GEO. Forty-seven GEO Series Experiments (GSEs) were found (Figure 1). A number of datasets were excluded due to duplication (*n* = 4), a different disease (*n* = 8), a lack of brain temporal region tissue samples (*n* = 28), a lack of mRNA expression data (*n* = 1), and a lack of a control (*n* = 2). The four datasets of GSE109887, GSE118553, GSE132903, and GSE138260 were selected for the MetaQC analysis. The data were divided according to the region of brain tissue, EC, MTG, and TC, if the gene expression data were measured from different brain tissues. A detailed description of these datasets is presented in Supplementary Table 1. Supplementary Table 2 shows the QC measures and standardized mean rank scores. The PCA plot is shown in Supplementary Figure 1. Although the first two PCs explained approximately 98% of the total variance, the datasets were scattered in the plot. Therefore, all four datasets were included in the meta-analysis, which contains 249 Alzheimer’s disease and 204 control lesions. Among the disease signatures, 74 genes including *ZNG621*, *ANTXR2*, and *DNALI1* showed increased expression levels in Alzheimer’s disease lesions compared to control lesions [log 2 (fold change) >1.0, adjusted *P* < 0.001], whereas 81 genes including *SYNGR1*, *TUBB2A*, *SVOP*, *STMN2*, and *KLHL35* showed decreased expression levels in dementia lesions [log 2 (fold change) <−1.0 adjusted *P* < 0.001] (Supplementary Figure 2). Sixty-two DEGs filtered *via* log 2 (fold change) >1.1 or <−1.1 and adjusted *P* < 0.001 are listed in Supplementary Table 3.

### Drug gene expression signatures

Reverse scores as drug signatures were computed according to changes in the expression levels of the 978 landmark genes from the LINCS data. These scores were computed based on 5,302 compound treatments in NPC human neural precursor cells. Summarized reverse scores were computed by weighting the concentrations and time points of the compounds and various cell lines. The minimal summarized reverse score was −1.014 and maximal summarized reverse scores was 1.093, respectively.

### Inhibitory concentrations in human brain tumor cells

**FIGURE 2:**
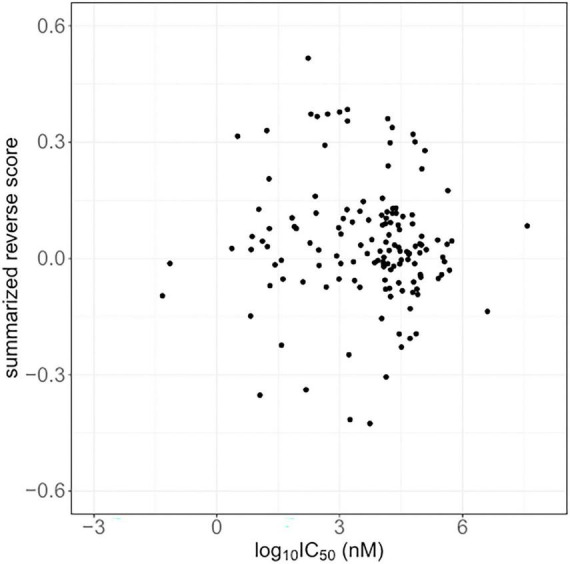
The summarized reverse gene expression scores and half-maximal inhibitory concentrations (IC50) from ChemBL.

The IC50 values for 875 compounds in human brain tumor cells, such as astrocytoma, glioblastoma, and neuroblastoma, were obtained from ChEMBL. The cell lines used for the IC50 values are listed in Supplementary Table 4. The IC50 values varied widely from 0.005 nM to 1320000 μM. Among the compounds, 174 compounds have computed reverse scores. Summarized reversal scores of the 174 compounds were variously distributed according to their median IC50 values (Figure 2).

### Reversed gene identification and compound predictions

**FIGURE 3:**
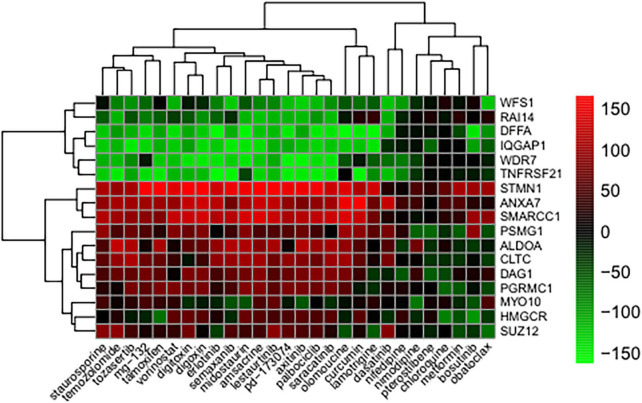
Genes whose expression was reversed in response to treatments with compound. The heatmap indicates the relative position of a gene in ranked compound expression data. Position are normalized and compound columns are ordered according to reversal scores. The distance matrix was calculated by the Euclidean method and the dendrogram was drawn by the complete linkage method. Low and high ranks suggest that the gene expression is down and upregulated, respectively, by the corresponding compound. Red and green colors indicate up and downregulation, respectively, after compound treatment.

The method of leave-one-out cross-validation was used to select reversely expressed genes by these compounds. Seventeen genes showed significantly reversed expressions (Table 1). *TNFRSF21*, *WDR7*, *DFFA*, *IQGAP1*, *WFS1*, *RAI14*, and *ANXA7* were downregulated upon treatment with compounds. *STMN1*, *SMARCC1*, *ALDOA*, *CLTC*, *PSMG1*, *SUZ12*, *DAG1*, *PGRMC1*, and *HMGCR*, and *MYO10* were upregulated following compound treatment Additionally, the compounds against Alzheimer’s disease were identified and their BBB permeability levels were predicted. The predicted BBB non-permeable compounds are listed in Supplementary Table 5, while the predicted BBB permeable compounds are shown in Figure 3. The protein kinase inhibitors including dasatinib, semaxanib, palbociclib, bosutinib, erlotinib, axitinib, tozasertib, saracatinib, lestaurtinib, staurosporine, and midostaurin, calcium channel blockers including nimodipine and nifedipine, histone deacetylase inhibitors including vorinostat, proteasome inhibitors including mg-132, and natural polyphenols including curcumin and pterostilbene were identified. Additionally, antineoplatic agents such as obatoclax, olomoucine, amsacrine, temozolomide, and tamoxifen were identified.

**TABLE 1:** Reversed genes expression by compounds in the Alzheimer’s disease.

Type*	Gene ID	Gene symbol	Description
Upregulated	27242	*TNFRSF21*	TNF receptor superfamily member 21
	23335	*WDR7*	WD repeat domain 7
	1676	*DFFA*	DNA fragmentation factor subunit alpha
	8826	*IQGAP1*	IQ motif containing GTPase activating protein 1
	7466	*WFS1*	Wolframin ER transmembrane glycoprotein
	26064	*RAI14*	Retinoic acid induced 14
	310	*ANXA7*	Annexin A7
Downregulated	3925	*STMN1*	Stathmin 1
	6599	*SMARCC1*	SWI/SNF related, matrix associated, actin dependent regulator of chromatin subfamily C member 1
	226	*ALDOA*	Aldolase, fructose-bisphosphate A
	1213	*CLTC*	Clathrin heavy chain
	8624	*PSMG1*	Proteasome assembly chaperone 1
	23512	*SUZ12*	SUZ12 polycomb repressive complex 2 subunit
	1605	*DAG1*	Dystroglycan 1
	10857	*PGRMC1*	Progesterone receptor membrane component 1
	3156	*HMGCR*	3-Hydroxy-3-methylglutaryl-CoA reductase
	4651	*MYO10*	Myosin X

*Up or downregulated means that the gene expression is up or downregulated after treatment.

## Discussion

The burden associated with Alzheimer’s disease continues to increase, and there is consequently an urgent need to seek innovative solutions to resolve this issue. In this study, we used a computational method that integrates disease-specific gene expression profiles and drug-induced gene expression profiles to predict drug candidates for use in Alzheimer’s disease treatments.

We identified seventeen therapeutic targets and twenty-eight repositioning candidates against Alzheimer’s disease. Several DEGs were found after a meta-gene expression analysis of Alzheimer’s disease in our study. It has been reported that amyloid precursor protein binds with tumor necrosis factor receptor superfamily member 21 (*TNFRSF21*) to induce neural inflammation in Alzheimer’s disease (Zhang et al., 2021). Induced wolframin endoplasmic reticulum transmembrane glycoprotein (*WFS1*) deficiency has been found to increase tau toxicity, which may play important roles in the development and progression of Alzheimer’s disease (Li et al., 2020). Progesterone receptor membrane component 1 (*PGRMC1*) increases the neuronal toxicity of amyloid beta-peptides by binding to the amyloid beta oligomer in Alzheimer’s disease (Qin et al., 2015). 3-Hydroxy-3-methylglutaryl-CoA reductase (*HMGCR*) was found to be a genetic modifier of the risk of Alzheimer’s disease (Leduc et al., 2015). Stathmin 1 (*STMN1*) as a cytosolic phosphoprotein that regulated microtubules dynamics, impaired axonal transport, and cause human neurodegenerative diseases (Duncan et al., 2013). Astrocytic dystrophin-associated complex components including dystroglycan 1 (*DAG1*) is known to be associated with temporal tau pathology (Simon et al., 2018).

Several repositionable candidate drugs, especially anticancer agents, against Alzheimer’s disease were identified in our study. Autophagy is the key mechanism by which to remove cellular abnormal protein aggregates, and the dysfunction of this process contributes to the pathogenesis of Alzheimer’s disease (Zhang et al., 2021). Most anticancer agents are able to induce autophagy or apoptosis, which may contribute to the neurodegenerative process (Eshraghi et al., 2022). Accumulating evidence has suggested that Alzheimer’s disease and cancer share some familiar biological pathways (Advani et al., 2020). Preclinical and clinical evaluations of several kinase inhibitors are ongoing in relation to Alzheimer’s disease (Fagiani et al., 2020; Prins et al., 2021). A pilot clinical trial of a senolytic combination therapy of dasatinib plus quercetin in early-stage Alzheimer’s disease was conducted (Gonzales et al., 2022). Additionally, vorinostat, a histone deacetylase inhibitor has also been proposed for use in the treatment of Alzheimer’s disease, phase I clinical trials were conducted (Eshraghi et al., 2022). However, it is important to consider that anticancer drugs can destroy subcellular components and disable fundamental biological processes. For instance, some anti-neoplastic agents can cause DNA damage, which is a critical pathological cause of Alzheimer’s disease (Lin et al., 2020). Therefore, we compared the reverse scores with the IC50 values of the compounds in our study. The reversal potency was not correlated to the IC50 values in Alzheimer’s disease, despite the higher variations in the reverse scores and IC50 values. It was previously reported that reverse scores were not correlated with IC50 values in cases involving microtubule inhibitors (Chen et al., 2017). The most commonly identified compound candidates in our results had microtubule-associated activity, and microtubule destabilization is known to be related to Alzheimer’s disease (Fernandez-Valenzuela et al., 2020). From this point of view, further investigations are required.

An antidiabetic agent, metformin (De Santi et al., 2019) exhibit pro-autophagy properties, which imply that these may be useful as a treatment for Alzheimer’s disease. Digoxin was reported to be beneficial in treating cognitive impairment in a mammalian model (Erdogan et al., 2022). The clinical efficacy of lamotrigine and changes in the dosages of concomitantly used psychotropic drugs were studied in relation to Alzheimer’s disease in conjunction with behavioral and psychological symptoms of dementia as a preliminary clinical trial (Suzuki and Gen, 2015). Calcium channel blockers have been reported to decrease significantly the rate of progression to dementia, which may minimize the formation of amyloid beta (Lovell et al., 2015), and nilvadipine was studied as a treatment in patients with mild to moderate Alzheimer’s disease (Lawlor et al., 2018).

Several natural products have been used in patients with Alzheimer’s disease. Curcumin has been shown to maintain the normal structure and function of cerebral vessels, mitochondria, and synapses effectively and to reduce the risk of Alzheimer’s disease (Chen et al., 2018). Numerous investigations in cellular and animal models have associated resveratrol and pterostilbene with protection against Alzheimer’s disease (Lange and Li, 2018).

On the other hand, with regard to repurposing for the treatment of Alzheimer’s disease, accessing the degree of penetration through the BBB requires significant consideration (Benn and Dawson, 2020). Therefore, we also predicted BBB permeability using deep learning and machine learning algorithms in our study.

Highly expressed *TNFRSF21* gene was significantly correlated with the dasatinib sensitivity or resistance (Huang et al., 2007). Nifedipine was found to activate 3-hydroxy-3-methyl-glutaryl-coenzyme A (HMG CoA) reductase, suggesting elevated production of cholesterol and phospholipids (Lin et al., 2019). *DFFA* gene associated with sensitivity or resistance of curcumin in tumor cells (Sertel et al., 2012) and treatment with curcumin was able to significantly increase the levels of *CLTC* mRNA in curcumin treated cells compared with control (Luo et al., 2018). The studies of association between candidate compounds and genes were still not enough for Alzheimer’s disease. The pathophysiologic mechanisms of Alzheimer’s disease are complex and are not well known. Pharmacological action and therapeutic outcomes cannot be determined by the simple approach of reverse gene expression profiling and the further evaluations need to replicate our results. Therefore, our findings likely indicate further investigations of treatments of Alzheimer’s disease. In summary, repositioning drug candidates and target genes in Alzheimer’s disease were identified using a computational method that combined disease-specific gene expressions with drug-induced gene expressions.

## Data availability statement

The datasets generated and/or analyzed for this study can be found are available from the corresponding author on reasonable request.

## Author contributions

I-WK analyzed the data and prepared the manuscript. HJ corrected and revised the manuscript. I-WK and JO contributed to the conception and design of the study. All authors were engaged in commenting on the manuscript, and read and approved the final manuscript.
