# Supplementary material for: Drug repurposing using meta-analysis of gene expression in Alzheimer’s disease
Source: Front Neurosci. 2022 Nov 10;16:989174. doi: 10.3389/fnins.2022.989174 (PMC9684643; doi:10.3389/fnins.2022.989174)
Supplement: Supplementary file 1 [file Data_Sheet_1.docx]

Supplementary Material

# Supplementary Table S1 Information about the gene expression datasets related to Alzheimer’s disease from the GEO

| GSE ID | Platform | Brain regions | Sample size (AD/control) |
| --- | --- | --- | --- |
| GSE109887 | Illumina HumanHT-12 V4.0 expression beadchip | Middle temporal gyrus | 46/32 |
| GSE118553^a^ | Illumina Human HT-12 V4.0 expression beadchip | Entorhinal cortex | 37/24 |
|  |  | Temporal cortex | 52/31 |
| GSE132903 | Illumina HumanHT-12 V4.0 expression beadchip | Middle temporal gyrus | 97/98 |
| GSE138260 | Agilent-034879 ADchip_1.0 033934 | Temporal cortex | 17/19 |

^a^The samples with asymptomatic Alzheimer’s disease were excluded. GEO, Gene Expression Omnibus; GSE, Gene Expression Omnibus Series Experiment; GPL, Gene Expression Omnibus platform; AD, Alzheimer’s disease

**Supplementary Table S2.** Quality control results using MetaQC

| GSE ID | IQC | EQC | CQCg | CQCp | AQCg | AQCp | Rank |
| --- | --- | --- | --- | --- | --- | --- | --- |
| GSE109887 | 5.80 | 2.42 | 307.65 | 289.42 | 183.65 | 126.21 | 1.58 |
| GSE118553_TC | 4.50 | 3.05 | 91.91 | 114.51 | 20.09 | 55.40 | 2.50 |
| GSE132903 | 3.79 | 1.73 | 307.65 | 240.69 | 103.03 | 99.88 | 2.58 |
| GSE118553_EC | 1.56* | 3.00 | 8.72 | 11.93 | 5.48 | 15.6 | 3.83 |
| GSE138260 | 2.56 | 2.46 | 0* | 7.2 | 0* | 1.53* | 4.50 |

GSE, Gene Expression Omnibus Series Experiments; IQC, Internal quality control; EQC, External quality control; CQCg, Consistency quality control in genes; CQCp, Consistency quality control in pathway, AQCg, Accuracy quality control in genes; AQCp, Accuracy quality control in pathways; EC, entorhinal cortex; TC, temporal cortex; Rank, a standardized mean rank; **P* value not significant after a Bonferroni correction

**Supplementary Table S3**. Disease gene expression signatures for Alzheimer’s disease

| GeneID | symbol | baseMean | log2(fold change) | *P* value  (×10^-20^) | Adjusted *P* value  (×10^-20^) |
| --- | --- | --- | --- | --- | --- |
| 71 | *ACTG1* | -1.145 | -1.104 | 1.00 | 3.24 |
| 165 | *AEBP1* | 1.141 | 1.185 | 1.00 | 3.24 |
| 79026 | *AHNAK* | 1.147 | 1.176 | 1.00 | 3.24 |
| 65124 | *ANKRD57* | 1.263 | 1.123 | 1.00 | 3.24 |
| 118429 | *ANTXR2* | 1.355 | 1.254 | 1.00 | 3.24 |
| 10476 | *ATP5H* | -1.090 | -1.179 | 1.00 | 3.24 |
| 527 | *ATP6V0C* | -1.160 | -1.156 | 1.00 | 3.24 |
| 529 | *ATP6V1E1* | -1.105 | -1.17 | 1.00 | 3.24 |
| 534 | *ATP6V1G2* | -1.203 | -1.223 | 1.00 | 3.24 |
| 633 | *BGN* | 1.050 | 1.102 | 1.00 | 3.24 |
| 388115 | *C15ORF52* | 0.989 | 1.132 | 1.00 | 3.24 |
| 8099 | *CDK2AP1* | 1.102 | 1.14 | 1.00 | 3.24 |
| 4166 | *CHST6* | 1.182 | 1.285 | 1.00 | 3.24 |
| 10491 | *CRTAP* | 1.224 | 1.142 | 1.00 | 3.24 |
| 1428 | *CRYM* | -1.184 | -1.187 | 1.00 | 3.24 |
| 115265 | *DDIT4L* | 1.115 | 1.117 | 1.00 | 3.24 |
| 7802 | *DNALI1* | 1.320 | 1.236 | 1.00 | 3.24 |
| 2026 | *ENO2* | -1.182 | -1.104 | 1.00 | 3.24 |
| 7430 | *EZR* | 1.040 | 1.18 | 1.00 | 3.24 |
| 23493 | *HEY2* | 0.979 | 1.104 | 1.00 | 3.24 |
| 3316 | *HSPB2* | 1.139 | 1.106 | 1.00 | 3.24 |
| 8988 | *HSPB3* | -1.018 | -1.114 | 1.00 | 3.24 |
| 8515 | *ITGA10* | 0.961 | 1.129 | 1.00 | 3.24 |
| 3693 | *ITGB5* | 1.183 | 1.153 | 1.00 | 3.24 |
| 3707 | *ITPKB* | 1.120 | 1.186 | 1.00 | 3.24 |
| 9764 | *KIAA0513* | -1.214 | -1.143 | 1.00 | 3.24 |
| 57613 | *KIAA1467* | -1.100 | -1.114 | 1.00 | 3.24 |
| 3831 | *KLC1* | -1.252 | -1.102 | 1.00 | 3.24 |
| 283212 | *KLHL35* | -1.271 | -1.118 | 1.00 | 3.24 |
| 1E+08 | *LOC100132707* | 1.234 | 1.228 | 1.00 | 3.24 |
| 266812 | *NAP1L5* | -1.010 | -1.134 | 1.00 | 3.24 |
| 4814 | *NINJ1* | 1.064 | 1.135 | 1.00 | 3.24 |
| 51299 | *NRN1* | -1.201 | -1.253 | 1.00 | 3.24 |
| 51559 | *NT5DC3* | -1.051 | -1.123 | 1.00 | 3.24 |
| 78991 | *PCYOX1L* | -1.097 | -1.118 | 1.00 | 3.24 |
| 5159 | *PDGFRB* | 1.132 | 1.201 | 1.00 | 3.24 |
| 51196 | *PLCE1* | 1.087 | 1.217 | 1.00 | 3.24 |
| 5364 | *PLXNB1* | 1.144 | 1.261 | 1.00 | 3.24 |
| 5613 | *PRKX* | 1.096 | 1.148 | 1.00 | 3.24 |
| 5725 | *PTBP1* | 1.026 | 1.106 | 1.00 | 3.24 |
| 284119 | *PTRF* | 1.023 | 1.111 | 1.00 | 3.24 |
| 6000 | *RGS7* | -1.046 | -1.109 | 1.00 | 3.24 |
| 23433 | *RHOQ* | 1.063 | 1.206 | 1.00 | 3.24 |

*(continued)*

| GeneID | symbol | baseMean | log2(fold change) | *P* value  (×10^-20^) | Adjusted *P* value  (×10^-20^) |
| --- | --- | --- | --- | --- | --- |
| 79685 | *SAP30L* | 1.136 | 1.181 | 1.00 | 3.24 |
| 51296 | *SLC15A3* | 1.185 | 1.191 | 1.00 | 3.24 |
| 6539 | *SLC6A12* | 1.061 | 1.125 | 1.00 | 3.24 |
| 6542 | *SLC7A2* | 1.018 | 1.137 | 1.00 | 3.24 |
| 23111 | *SPG20* | 1.157 | 1.124 | 1.00 | 3.24 |
| 80309 | *SPHKAP* | -1.031 | -1.113 | 1.00 | 3.24 |
| 10274 | *STAG1* | 1.020 | 1.176 | 1.00 | 3.24 |
| 11075 | *STMN2* | -1.171 | -1.121 | 1.00 | 3.24 |
| 55530 | *SVOP* | -1.213 | -1.196 | 1.00 | 3.24 |
| 85360 | *SYDE1* | 1.130 | 1.122 | 1.00 | 3.24 |
| 6853 | *SYN1* | -1.118 | -1.106 | 1.00 | 3.24 |
| 9145 | *SYNGR1* | -1.500 | -1.345 | 1.00 | 3.24 |
| 140597 | *TCEAL2* | -1.149 | -1.174 | 1.00 | 3.24 |
| 8463 | *TEAD2* | 1.166 | 1.224 | 1.00 | 3.24 |
| 129303 | *TMEM150A* | 1.060 | 1.191 | 1.00 | 3.24 |
| 7102 | *TSPAN7* | -1.180 | -1.194 | 1.00 | 3.24 |
| 7280 | *TUBB2A* | -1.205 | -1.232 | 1.00 | 3.24 |
| 220929 | *ZNF438* | 0.884 | 1.115 | 1.00 | 3.24 |
| 285268 | *ZNF621* | 1.437 | 1.216 | 1.00 | 3.24 |
| 6853 | *SYN1* | -1.118 | -1.106 | 1.00 | 3.24 |
| 9145 | *SYNGR1* | -1.500 | -1.345 | 1.00 | 3.24 |

Absolute values of log_2_(fold change)>1.1 and adjusted *P* value<0.001 were used to identify significantly differentially expressed genes between psoriatic and normal lesions.

**Supplementary Table S4**. The brain tumor cell lines used for IC50 values.

| Cancer type | Cell line name |
| --- | --- |
| Astrocytoma | Becker, CCF-STTG1, DBTRG-05MG, H4, KINGS1, NMC-G1, SF268, SNB-19, SW 1088, SW 1783, U-118 MG, U138 MG, U-251, U373 |
| Glioblastoma | 8-MG-BA, A172, AM-38, CAS-1, D263MG, D423MG, D542MG, DK-MG, GAMG, GB-1, GBM, GMS-10, KALS-1, KNS-42, KNS-81-FD, KS-1, LN-18, LN-229, LN405, M059J, NCH82, NCH89, NO10, NO-11, SF-126, SF-188, SF-295, SNB-7, SNB-75, SNB-78, T98G, TSGH, U3013MG, U-87 MG, XF498, YH-13, YKG-1 |
| Neuroblastoma | CAN, CHP-126, CHP-212, GI-ME-N, GOTO, IMR-5, IMR-32, IMR-5/75, KELLY, KP-N-RT-BM-1, KP-N-YN, KP-N-YS, LAN-6, MHH-NB-11, NB1, NB69, NBsusSR, NH-12, NH-6, SCCH-26, SH-SY5Y, SIMA, SK-N-AS, BE(2)-C, SK-N-BE(2)-M1, SK-N-DZ, SK-N-FI, SK-N-SH, TGW |
| Medulloblastoma | Daoy, ONS-76 |
| Oligodendroglioma | Hs 683 |
| Gliosarcoma | D247MG, GI-1, NCH37, SF539 |

**Supplementary Table S5**. The predicted BBB non-permeable compounds using DeePred-BBB.

| Compound | Canonical SMILES |
| --- | --- |
| Afatinib | CN(C)CC=CC(=O)NC1=C(C=C2C(=C1)C(=NC=N2)NC3=CC(=C(C=C3)F)Cl)OC4CCOC4 |
| AZD-6482 | CC1=CN2C(=O)C=C(N=C2C(=C1)C(C)NC3=CC=CC=C3C(=O)O)N4CCOCC4 |
| Betulinic acid | CC(=C)C1CCC2(C1C3CCC4C5(CCC(C(C5CCC4(C3(CC2)C)C)(C)C)O)C)C(=O)O |
| Celecoxib | CC1=CC=C(C=C1)C2=CC(=NN2C3=CC=C(C=C3)S(=O)(=O)N)C(F)(F)F |
| Cholic acid | CC(CCC(=O)O)C1CCC2C1(C(CC3C2C(CC4C3(CCC(C4)O)C)O)O)C |
| Crizotinib | CC(C1=C(C=CC(=C1Cl)F)Cl)OC2=C(N=CC(=C2)C3=CN(N=C3)C4CCNCC4)N |
| Cyclopamine | CC1CC2C(C(C3(O2)CCC4C5CC=C6CC(CCC6(C5CC4=C3C)C)O)C)NC1 |
| Docetaxel | CC1=C2C(C(=O)C3(C(CC4C(C3C(C(C2(C)C)(CC1OC(=O)C(C(C5=CC=CC=C5)NC(=O)OC(C)(C)C)O)O)OC(=O)C6=CC=CC=C6)(CO4)OC(=O)C)O)C)O |
| Entinostat | C1=CC=C(C(=C1)N)NC(=O)C2=CC=C(C=C2)CNC(=O)OCC3=CN=CC=C3 |
| Epirubicin | CC1C(C(CC(O1)OC2CC(CC3=C2C(=C4C(=C3O)C(=O)C5=C(C4=O)C(=CC=C5)OC)O)(C(=O)CO)O)N)O |
| Etoposide | CC1OCC2C(O1)C(C(C(O2)OC3C4COC(=O)C4C(C5=CC6=C(C=C35)OCO6)C7=CC(=C(C(=C7)OC)O)OC)O)O |
| Floxuridine | C1C(C(OC1N2C=C(C(=O)NC2=O)F)CO)O |
| Geldanamycin | CC1CC(C(C(C=C(C(C(C=CC=C(C(=O)NC2=CC(=O)C(=C(C1)C2=O)OC)C)OC)OC(=O)N)C)C)O)OC |
| Genistein | C1=CC(=CC=C1C2=COC3=CC(=CC(=C3C2=O)O)O)O |
| Indirubin | C1=CC=C2C(=C1)C(=C(N2)O)C3=NC4=CC=CC=C4C3=O |
| LY-294002 | C1COCCN1C2=CC(=O)C3=C(O2)C(=CC=C3)C4=CC=CC=C4 |
| Motesanib | CC1(CNC2=C1C=CC(=C2)NC(=O)C3=C(N=CC=C3)NCC4=CC=NC=C4)C |
| Niclosamide | C1=CC(=C(C=C1[N+](=O)[O-])Cl)NC(=O)C2=C(C=CC(=C2)Cl)O |
| Paclitaxel | CC1=C2C(C(=O)C3(C(CC4C(C3C(C(C2(C)C)(CC1OC(=O)C(C(C5=CC=CC=C5)NC(=O)C6=CC=CC=C6)O)O)OC(=O)C7=CC=CC=C7)(CO4)OC(=O)C)O)C)OC(=O)C |
| Pazopanib | CC1=C(C=C(C=C1)NC2=NC=CC(=N2)N(C)C3=CC4=NN(C(=C4C=C3)C)C)S(=O)(=O)N |
| Plumbagin | CC1=CC(=O)C2=C(C1=O)C=CC=C2O |
| PLX-4720 | CCCS(=O)(=O)NC1=C(C(=C(C=C1)F)C(=O)C2=CNC3=C2C=C(C=N3)Cl)F |
| Resveratrol | C1=CC(=CC=C1C=CC2=CC(=CC(=C2)O)O)O |
| SB-216763 | CN1C=C(C2=CC=CC=C21)C3=C(C(=O)NC3=O)C4=C(C=C(C=C4)Cl)Cl |
| Securinine | C1CCN2C(C1)C34CC2C=CC3=CC(=O)O4 |
| Sitagliptin | C1CN2C(=NN=C2C(F)(F)F)CN1C(=O)CC(CC3=CC(=C(C=C3F)F)F)N |
| Sunitinib | CCN(CC)CCNC(=O)C1=C(NC(=C1C)C=C2C3=C(C=CC(=C3)F)NC2=O)C |
| Tacrolimus | CC1CC(C2C(CC(C(O2)(C(=O)C(=O)N3CCCCC3C(=O)OC(C(C(CC(=O)C(C=C(C1)C)CC=C)O)C)C(=CC4CCC(C(C4)OC)O)C)O)C)OC)OC |
| Tanespimycin | CC1CC(C(C(C=C(C(C(C=CC=C(C(=O)NC2=CC(=O)C(=C(C1)C2=O)NCC=C)C)OC)OC(=O)N)C)C)O)OC |

*(continued)*

| Compound | Canonical SMILES |
| --- | --- |
| Topotecan | CCC1(C2=C(COC1=O)C(=O)N3CC4=CC5=C(C=CC(=C5CN(C)C)O)N=C4C3=C2)O |
| Torin-1 | CCC(=O)N1CCN(CC1)C2=C(C=C(C=C2)N3C(=O)C=CC4=CN=C5C=CC(=CC5=C43)C6=CC7=CC=CC=C7N=C6)C(F)(F)F |
| Tretinoin | CC1=C(C(CCC1)(C)C)C=CC(=CC=CC(=CC(=O)O)C)C |
| Vinblastine | CCC1(CC2CC(C3=C(CCN(C2)C1)C4=CC=CC=C4N3)(C5=C(C=C6C(=C5)C78CCN9C7C(C=CC9)(C(C(C8N6C)(C(=O)OC)O)OC(=O)C)CC)OC)C(=O)OC)O |
| Vincristine | CCC1(CC2CC(C3=C(CCN(C2)C1)C4=CC=CC=C4N3)(C5=C(C=C6C(=C5)C78CCN9C7C(C=CC9)(C(C(C8N6C=O)(C(=O)OC)O)OC(=O)C)CC)OC)C(=O)OC)O |
| Vinorelbine | CCC1=CC2CC(C3=C(CN(C2)C1)C4=CC=CC=C4N3)(C5=C(C=C6C(=C5)C78CCN9C7C(C=CC9)(C(C(C8N6C)(C(=O)OC)O)OC(=O)C)CC)OC)C(=O)OC |
| VX-702 | C1=CC(=C(C(=C1)F)N(C2=NC(=C(C=C2)C(=O)N)C3=C(C=C(C=C3)F)F)C(=O)N)F |


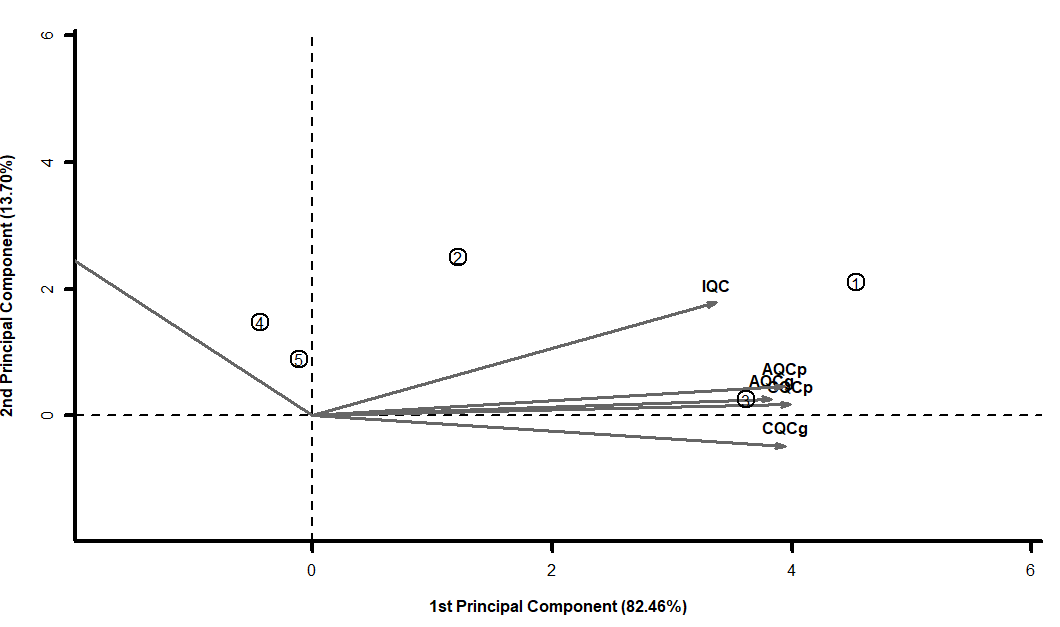


**Supplementary Figure S1.** Principle component analysis for quantitative quality control. Each circled number represents the overall rank by a standardized mean rank summary score of a study. Smaller numbers correspond to higher quality studies. The first principal component is presented on the x-axis, while the second principle component is shown on the y-axis. MetaQC used for accuracy quality control in genes (AQCg), accuracy quality control in pathway (AQCp), consistency quality control in genes (CQCg), consistency quality control in pathway (CQCp), external quality control (EQC) and internal quality control (IQC) measures. EC, entorhinal cortex; TC, temporal cortex 1, GSE109887; 2, GSE118553_EC; GSE132903; 3, GSE118553_TC; 7, GSE138260


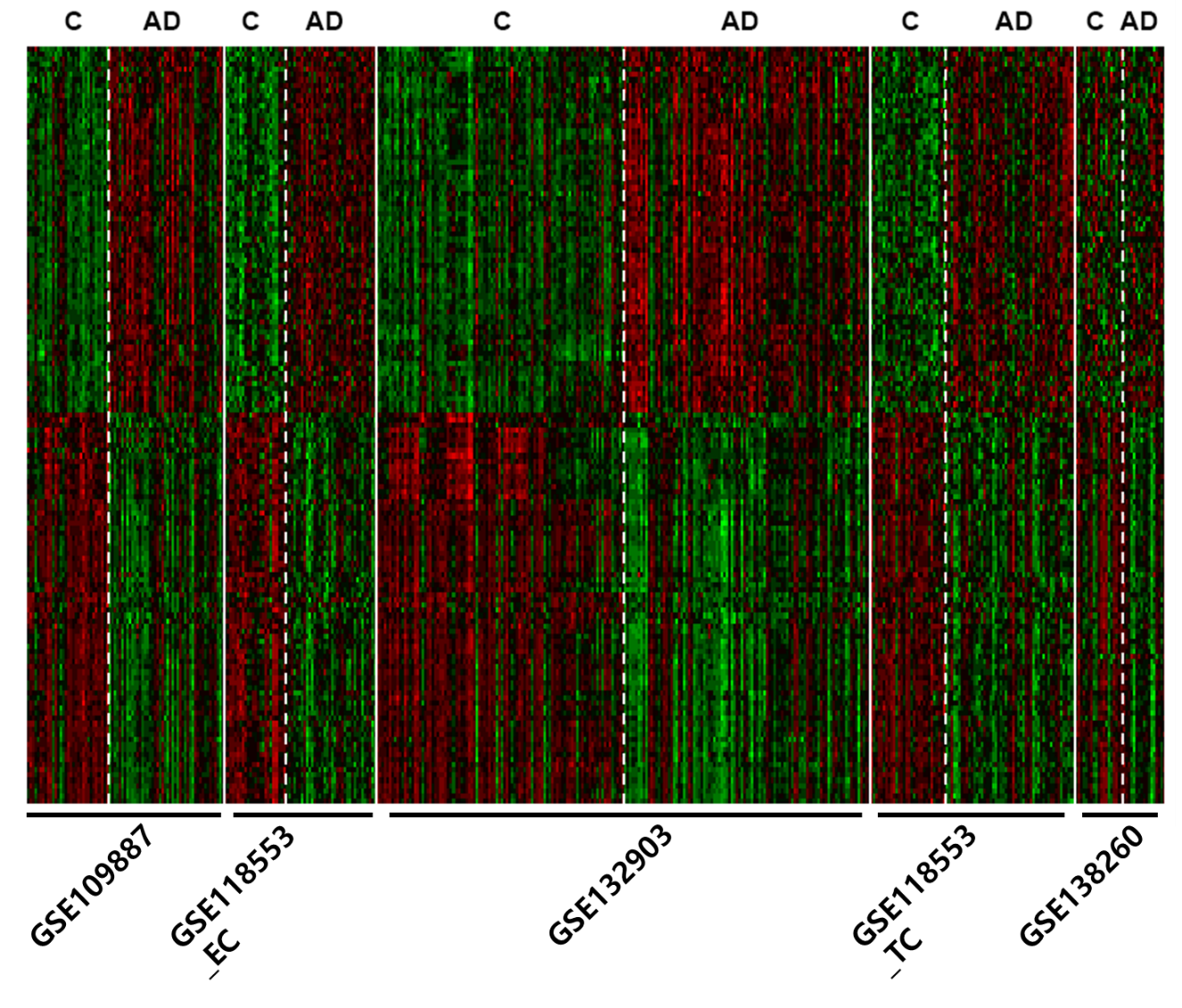


**Supplementary Figure S2**. Heatmap of expression profiles of the top up- and downregulated differentially expressed genes from the meta-analysis of Alzheimer’s disease. Genes with log2fold changes exceeding 1.0 with the false discovery rate set to <0.001: upregulated genes shown in red; downregulated genes shown in green. C, control; AD, Alzheimer’s disease, GSE, Gene Expression Omnibus Series Experiments; EC, entorhinal cortex; TC, temporal cortex
